# Supplementary material for: Ultra-Rapid serial visual presentation reveals dynamics of feedforward and feedback processes in the ventral visual pathway
Source: eLife. 2018 Jun 21;7:e36329. doi: 10.7554/eLife.36329 (PMC6029845; doi:10.7554/eLife.36329)
Supplement: Transparent reporting form [file elife-36329-transrepform.pdf]

eLIFE
1st Floor
24 Hills Road
Cambridge CB2 1JP, UK
P 01223 855340
W elifesciences.org
T @elife

eLife's transparent reporting form

We encourage authors to provide detailed information *within their submission* to facilitate the interpretation and replication of experiments. Authors can upload supporting documentation to indicate the use of appropriate reporting guidelines for health-related research (see EQUATOR Network), life science research (see the BioSharing Information Resource), or the ARRIVE guidelines for reporting work involving animal research. Where applicable, authors should refer to any relevant reporting standards documents in this form.

If you have any questions, please consult our Journal Policies and/or contact us: editorial@elifesciences.org.

Sample-size estimation

- You should state whether an appropriate sample size was computed when the study was being designed
- You should state the statistical method of sample size computation and any required assumptions
- If no explicit power analysis was used, you should describe how you decided what sample (replicate) size (number) to use

Please outline where this information can be found within the submission (e.g., sections or figure legends), or explain why this information doesn't apply to your submission:

We report sample size information in the Materials and Methods section. A total of n=17 healthy subjects with normal or corrected to normal vision participated in the MEG RSVP experiment. The fMRI data set consisted of recordings from n=15 healthy participants and was part of a larger 92-image data set that has been previously published (Cichy et al., 2014). We reported the sample size throughout the manuscript whenever we presented statistical results, including the figure captions and the main text. Our sample size was chosen based on several prior multivariate decoding studies from our group and others that have applied similar analytical methods (e.g. Cichy et al., 2014; Isik et al., 2014; Salti et al., 2015; sample sizes in the range of 11-17).

Replicates

- You should report how often each experiment was performed
- You should include a definition of biological versus technical replication
- The data obtained should be provided and sufficient information should be provided to indicate the number of independent biological and/or technical replicates
- If you encountered any outliers, you should describe how these were handled
- Criteria for exclusion/inclusion of data should be clearly stated
- High-throughput sequence data should be uploaded before submission, with a private link for reviewers provided (these are available from both GEO and ArrayExpress)

Please outline where this information can be found within the submission (e.g., sections or figure legends), or explain why this information doesn't apply to your submission:

eLife Sciences Publications, Ltd is a limited liability non-profit non-stock corporation incorporated in the State of Delaware, USA, with company number 5030732, and is registered in the UK with company number FC030576 and branch number BR015634 at the address 1st Floor, 24 Hills Road, Cambridge CB2 1JP | August 2014

As we reported in Material and Methods we included the data of all n=17 subjects from the MEG RSVP experiment. Collecting data from 17 subjects is a biological replicate. The fMRI data with n=15 subjects were published previously in Cichy et al. 2014. We followed the following practices for data inclusion/exclusion: A 6000 fT peak-to-peak rejection threshold was set to discard bad MEG trials. To avoid eye movement artifacts, the subjects were asked to fixate on a black cross presented at the center of the screen and blink only when pressing a button and not during the RSVP sequences. Eye blink artifacts were automatically detected from frontal sensor MEG data, and then principal component analysis was used to remove these artifacts.

Statistical reporting

- Statistical analysis methods should be described and justified
- Raw data should be presented in figures whenever informative to do so (typically when N per group is less than 10)
- For each experiment, you should identify the statistical tests used, exact values of N, definitions of center, methods of multiple test correction, and dispersion and precision measures (e.g., mean, median, SD, SEM, confidence intervals; and, for the major substantive results, a measure of effect size (e.g., Pearson's r, Cohen's d)
- Report exact p-values wherever possible alongside the summary statistics and 95% confidence intervals. These should be reported for all key questions and not only when the p-value is less than 0.05.

Please outline where this information can be found within the submission (e.g., sections or figure legends), or explain why this information doesn't apply to your submission:

The information about statistical inference can be found in the Material and Methods section under "Statistical Inference".

Statistical inference relied on non-parametric statistical tests that do not make assumptions on the distributions of the data (Maris and Oostenveld, 2007; Pantazis et al., 2005). Specifically, for the statistical assessment of classification time series, temporal generalization matrices, and MEG-fMRI representational similarities we performed permutation-based cluster-size inference. The null hypothesis was equal to 50% chance level for decoding results, and 0 for decoding differences or correlation values. In all cases we could permute the condition labels of the MEG data, which was equivalent to a sign permutation test that randomly multiplied subject responses by +1 or -1. We used 1000 permutations, 0.05 cluster defining threshold and 0.05 cluster threshold for time series and temporal generalization maps; and 0.001 cluster defining threshold and 0.05 cluster threshold for 4-dimensional maps.

For statistical assessment of peak latency of the time series, we performed bootstrap tests. The subject-specific time series were bootstrapped 1000 times and the empirical distribution of the peak latency of the subject-averaged time series was used to define 95% confidence intervals. For peak-to-peak latency differences, we obtained 1000 bootstrapped samples of the difference between the two peaks, and we rejected the null hypothesis of no peak-to-peak latency difference if the 95% confidence interval did not include 0.

eLife Sciences Publications, Ltd is a limited liability non-profit non-stock corporation incorporated in the State of Delaware, USA, with company number 5030732, and is registered in the UK with company number FC030576 and branch number BR015634 at the address 1st Floor, 24 Hills Road, Cambridge CB2 1JP | August 2014

(For large datasets, or papers with a very large number of statistical tests, you may upload a single table file with tests, Ns, etc., with reference to sections in the manuscript.)

Group allocation

- Indicate how samples were allocated into experimental groups (in the case of clinical studies, please specify allocation to treatment method); if randomization was used, please also state if restricted randomization was applied
- Indicate if masking was used during group allocation, data collection and/or data analysis

Please outline where this information can be found within the submission (e.g., sections or figure legends), or explain why this information doesn't apply to your submission:

The details can be found in Materials and Methods under "Experimental Design and Stimulus Set". Our study included one experimental group (n=17 subjects recruited from the local community for the RSVP MEG experiment). Each subject was exposed to the same experimental conditions in a repeated-measures design. A different cohort of n=15 participants participated in the fMRI experiment and 1.5 s MEG experiment, which was previously published in Cichy et al., 2014.

Additional data files ("source data")

- We encourage you to upload relevant additional data files, such as numerical data that are represented as a graph in a figure, or as a summary table
- Where provided, these should be in the most useful format, and they can be uploaded as "Source data" files linked to a main figure or table
- Include model definition files including the full list of parameters used
- Include code used for data analysis (e.g., R, MatLab)
- Avoid stating that data files are "available upon request"

Please indicate the figures or tables for which source data files have been provided:

We provide data and analysis MATLAB scripts to reproduce the figures and main findings of the article.
